# Identification of Gene Biomarkers for Distinguishing Small-Cell Lung Cancer from Non-Small-Cell Lung Cancer Using a Network-Based Approach

**DOI:** 10.1155/2015/685303

**Published:** 2015-07-28

**Authors:** Fei Long, Jia-Hang Su, Bin Liang, Li-Li Su, Shu-Juan Jiang

**Affiliations:** ^1^Department of Respiratory Medicine, Shandong Provincial Hospital Affiliated to Shandong University, Jinan, Shandong 250021, China; ^2^Department of Pharmacy, Yantai Chinese Medicine Hospital, Yantai, Shandong 264100, China

## Abstract

Lung cancer consists of two main subtypes: small-cell lung cancer (SCLC) and non-small-cell lung cancer (NSCLC) that are classified according to their physiological phenotypes. In this study, we have developed a network-based approach to identify molecular biomarkers that can distinguish SCLC from NSCLC. By identifying positive and negative coexpression gene pairs in normal lung tissues, SCLC, or NSCLC samples and using functional association information from the STRING network, we first construct a lung cancer-specific gene association network. From the network, we obtain gene modules in which genes are highly functionally associated with each other and are either positively or negatively coexpressed in the three conditions. Then, we identify gene modules that not only are differentially expressed between cancer and normal samples, but also show distinctive expression patterns between SCLC and NSCLC. Finally, we select genes inside those modules with discriminating coexpression patterns between the two lung cancer subtypes and predict them as candidate biomarkers that are of diagnostic use.

## 1. Introduction

Lung cancer is the most commonly occurring type of cancers worldwide. In China, lung cancer has become the first cause of cancer death in China, with the fastest rising mortality in population [1]. According to the third nationwide Sampling Survey of Death Cause Review conducted by China's Ministry of Health in 2006, the mortality caused by lung cancer has increased by 75.77% since 1990s and has increased by 33.25% after excluding factors of age structure changes. Although it has been widely recognized that smoking is the most related risk factor for lung cancer [2], the complete understanding of pathogenesis, disease diagnostics, and the development of therapy of lung cancer are still under active research.

Lung cancer consists of two major histological types: small-cell lung cancer (SCLC) and non-small-cell lung cancer (NSCLC) [3]. SCLC is defined as “a malignant epithelial tumor consisting of small cells with scant cytoplasm, ill-defined cell borders, finely granular nuclear chromatin, and absent or inconspicuous nucleoli” [4]. Though SCLC is not very common in lung cancers (about 20%), it has a strong relationship with smoking and has a rapid growth rate, early metastases, and high initial response rates [5]. It usually arises from major bronchi centrally, with extensive mediastinal adenopathy [3]. NSCLC includes squamous-cell (epidermoid) carcinoma, adenocarcinoma, and large-cell carcinoma. Adenocarcinoma and large-cell carcinoma usually arise from the small bronchi, bronchioles, or alveoli (peripheral tumors) of the distant airway of the lung peripherally, whereas squamous-cell carcinoma usually arise centrally [6]. Squamous-cell carcinoma is characterized by lobar collapse, obstructive pneumonia, or hemoptysis and shows late development of distant metastases. Adenocarcinoma is associated with early development of metastases, with some of the primary tumors as a symptomless peripheral lesion. Large-cell carcinoma has large peripheral masses, sometimes with cavitation [3].

Lung cancer patients usually have no symptoms or have nonspecific symptoms such as shortness of breath, coughing, and weight loss at the early stage of cancer development, making early diagnosis of lung cancer extremely difficult. Most diagnosed lung cancer patients are at their late stage of cancer development, which is the main reason for the high mortality rate of lung cancer. It is therefore of dire need to develop sensitive and reliable tools for diagnosis of lung cancer. Usually, the diagnosis of lung cancer involves (i) the identification and complete classification of malignancy, (ii) immunohistochemistry to distinguish lung cancer subtypes, and (iii) molecular testing [7]. Given that different subtypes of lung cancer have significantly different responsiveness to treatment, for example, SCLC usually responds better to chemotherapy and radiotherapy, whereas NSCLC patients often have to be treated with surgery [8], in this study we focus on developing method for distinguishing different types of lung cancer that can be of diagnostic use.

During the development and progression of cancer, there are significant genetic and molecular alterations in cells, which can be permanent, irreversible, and dynamic and cause significant variations in gene expression [9]. Thus, detecting genes with signature expression change can provide diagnosis markers. For instance, Zhang et al. analyzed the genome-wide expression profiles of both mRNAs and miRNAs in three NSCLC cell lines and identified hundreds of genes and 10 miRNAs with significant expression changes that can be used as potential diagnosis markers [10]. Mishra et al. investigated the differential gene expression profile in different lung cancer cell line and discovered a number of genes whose expression is significantly correlated with patients' survival [11]. Here, we aim to identify genes with significant gene expression change between the two subtypes of lung cancer. This could be done by simply comparing the expression level of a gene between the two subtypes of lung cancer. However, mounting evidence has suggested that this is not a reliable approach. First, this approach is not robust and is significantly biased by expression noise [12]. Second, the top differentiated expressed genes may not be the causal genes but the downstream response genes. Third, in cell genes do not function by themselves but in a complicated network, and ignoring the relationships between genes will significantly reduce the power of finding the truly important genes. Thus, we attempt to tackle the problem by integrating gene expression profiles with protein-protein interaction information using a network-based approach. We first collect gene expression datasets from GEO, including normal lung tissue, SCLC, and NSCLC [13–15]. Then, after determining gene pairs with significant positive or negative correlation in gene expression in each of the three types of datasets, we map those gene pairs to the STRING network [16], a large-scale gene functional association network, and then construct a lung cancer-specific functional association network. We further partition this network into gene modules and identify the modules with significant association with either SCLC or NSCLC in terms of gene expression variation. These gene modules are considered to be potentially useful for distinguishing SCLC from NSCLC. Functional enrichment analysis has revealed that those gene modules are highly related to lung cancer development. Finally, from the gene modules we identify genes with specific association with either SCLC or NSCLC. These genes can be exploited as potential biomarkers of diagnostic use for distinguishing SCLC or NSCLC.

## 2. Methods and Materials

### 2.1. Dataset Collection and Processing

We use GEO query [17] to download gene expression datasets from GEO database [18] and prepare three datasets corresponding to normal lung tissue, SCLC, and NSCLC, respectively (Table 1). These three datasets are selected using the following rules: (1) the samples must be human tissue samples; (2) the sample size should be greater than 100; and (3) there is no special treatment. Since SCLC is not common, we reduce the sample size to be more than 20. We use R package limma [19] for gene expression normalization. Then, for each gene expression dataset we calculate Pearson Correlation Coefficient (PCC) for each pair of genes using their expression profile and select the top 1% and bottom 1% gene pairs as positively or negatively coexpressed genes, respectively. These positively or negatively coexpression gene pairs from the three datasets are combined together and are mapped to the STRING [16] network. These mapped gene pairs with a functional association score greater than 500 in the STRING network are retained and are used to construct a lung cancer-specific functional association network. This network is then partitioned into gene modules using a network partition algorithm called iNP [20]. For validation purpose, we also prepare a new dataset for NSCLC (GSE10245 [21]) whose sample size is 58 and repeat the above procedures to construct a new lung cancer-specific functional association network.

### 2.2. Identification of Lung Cancer-Specific Gene Modules

For each of the partitioned gene modules, we test whether it has significant gene expression variation between normal, SCLC, and NSCLC samples. Since each module includes a group of genes that are significantly functionally related to each other, a module can be considered as a pathway. There are many ways to determine whether a pathway is significantly differentially expressed between cancer and normal samples. Here, we choose a simple method, the median expression value. We use the median expression value for all genes inside the module as the representative expression value for the module in a given sample. Then, we perform* t*.test to compare the module's expression values in either SCLC or NSCLS samples against those in normal samples and define the differentially expressed modules between lung cancer and normal samples as those with *P* value < 0.01 (adjusted by FDR) and the log (fold change) greater than 2. Next, from those differentially expressed modules we further quantitatively determine their specificity in distinguishing SCLC or NSCLC by plotting a ROC curve using the module's expression value in SCLC samples against the values in NSCLC samples. The AUC (area under curve) of the ROC curve is calculated and is used to indicate the specificity of the module to either SCLC or NSCLC. The AUC ranges from 0 to 1, with random association equaling to 0.5. AUC of 0 or 1 corresponds to perfect specific in distinguishing the two lung cancer subtypes. In addition, if the AUC is greater than 0.5, it indicates that the module tends to be upregulated in SCLC compared to that in NSCLC or vice versa. We use Cytoscape 3.1 to display module structure and use R to draw clustering figures.

### 2.3. Function Enrichment Analysis

Given a selected gene module, we perform function enrichment for the genes inside the module. GO annotation file is downloaded from [22] on Nov. 23rd, 2014. Pathway annotation from MSigDB is downloaded from GSEA [23]. Biological process GO terms and MSigDB pathways are tested for enrichment using Fisher's test() in R. The significance of threshold was set at 0.01. Since there are many enriched gene sets that are highly overlapping (sharing common genes), we use a cluster-and-filter strategy to reduce the enriched gene sets. In the cluster-step, we first calculate the relatedness between all gene sets (defined as the number of overlapped genes/the number of union genes) and form a gene set-based network. Then, we use iNP to partition the network into modules within which gene sets are highly overlapping. In the filter step, after the enrichment analysis, we map all enriched gene sets to gene set modules and then select the most significantly enriched gene set within each module as the representative gene sets. Finally, we collect all representative gene sets of each enriched module to form a reduced list of enriched gene sets.

### 2.4. Detection of Genes with Significantly Different Expression Pattern between the Stages and Subtypes of NSCLC

The NSCLC datasets provide the stage and subtype information for each sample. By grouping samples according to stages or subtypes, we perform ANOVA test to determine whether a given gene's expression value is significantly different in at least one stage or subtype of NSCLC. We set *P* value threshold as 0.05 with fdr adjustment.

## 3. Results

### 3.1. Construction of a Lung Cancer-Specific Functional Association Network

We prepare three gene expression datasets for normal lung tissues (1,349 samples), SCLC (90 samples), and NSCLC (275 samples), respectively, from GEO database (Table 1). For each dataset, we calculate Pearson Correlation Coefficient (PCC) for each pair of genes using their expression profiles in the datasets. PCC ranges from −1 to 1, with 1 indicating positive correlation and −1 indicating negative correlation. After sorting all gene pairs according to their PCC values, from each of the three datasets we select the top 1% and the bottom 1% gene pairs as the positively and negatively coexpressed gene pairs. Then, we combine all gene pairs selected from the three datasets and map them to the STRING network [16], a functional association network. By retaining those gene pairs that have a functional association score greater than 500 (the score indicates a strong functional association between the pair of genes), we obtain a lung cancer-specific gene functional association network, which is a binary network, consisting of 7,572 genes and 43,816 edges.

### 3.2. Identification of Differentially Expressed Gene Modules in Lung Cancer

**Figure 1:**
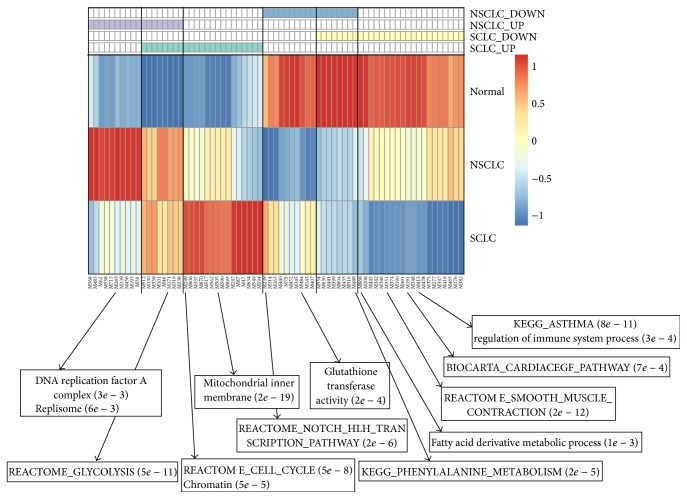
Heatmap of gene modules that have differential expression pattern between SCLC or NSCLC and normal lung tissue samples. Each column represents a module. The top four rows represent the types of differential expression pattern. For example, SCLC_up means the module is upregulated in SCLC, compared to normal tissues. The most significantly enriched functions of selected gene modules are shown with *P* values at the bottom of the heatmap.

Using a network partition algorithm called iNP [20], we partition the lung cancer-specific gene functional association network into 737 modules with a modularity of 0.53. Genes within each module are highly functionally associated with each other. To determine whether a gene module is differentially expressed between cancer and normal samples, we first determine the expression value of the module using the median expression value of genes inside the module in each sample. Then, similar to determining differentially expressed genes, we use* t*.test to identify modules that are differentially expressed between SCLC or NSCLC samples and normal lung tissues samples. We find a total number of 71 modules that are significantly differentiated expressed between lung cancer and normal tissues (Figure 1). Of these modules, 23 and 28 are significantly up- and downregulated in SCLC, respectively. As for NSCLC, there are 18 upregulated and 18 downregulated modules. In addition, there are 8 modules that are upregulated in both SCLC and NSCLC, and 8 modules that are downregulated in both SCLC and NSCLC.

### 3.3. Functional Analysis of Differentially Expressed Gene Modules in Lung Cancer

To obtain functional insights about the differentially expressed gene modules, we conduct pathway enrichment analysis for genes inside those modules. The full list of the module ID, genes inside the module, and enriched functions can be found in Supplemental Table 1 in Supplementary Material available online at http://dx.doi.org/10.1155/2015/685303. Here, we only provide a reduced list of enriched functions (see Methods for details) in order to reduce the redundancy among enriched functions. The most significantly enriched functions of selected gene modules are shown in Figure 1 for illustrative purpose. For gene modules that are upregulated in both SCLC and NSCLC, some of them are enriched with glycolysis metabolic process, a common hallmark for many types of cancers [24]. For the gene modules that are only upregulated in SCLC, we find that their functions are involved in mitochondrial, cell cycle, and chromatin organization. This is consistent with the phenotypic description of SCLC, which says that cancer cells of SCLC have finely granular nuclear chromatin, suggesting that chromatin organization may be disrupted. It has also been proposed that a therapeutic strategy for SCLC can be to target the mitochondrial apoptosis pathway [5]. For gene modules that are downregulated in only SCLC, they are enriched with diverse functions, such as asthma, regulation of immune system process, muscle contraction, and fatty acid metabolism. Though asthma is rarely related to SCLC, some cases have been reported [25]. Fatty acids in erythrocyte are treated as potential biomarkers in the diagnosis of lung cancer [26]. For gene modules that are upregulated in only NSCLC, some are enriched with function involved in DNA replication, which is consistent with previous findings that genes DNA repair are closely related to the risk of NSCLC [27]. For gene modules downregulated only in NSCLC, some of them are enriched with Notch pathway and glutathione transferase activity, both of which have been reported to be strongly related to lung cancer [28, 29]. Thus, functional analysis of differentially expressed genes supports that these gene modules are strongly related to lung cancer development, making it possible for us to conduct further exploitation to identify gene biomarkers that can be of diagnosis use for distinguishing the two subtypes of lung cancer.

### 3.4. Identification of Potential Diagnosis Biomarkers that Distinguish SCLC and NSCLC

Given the 71 gene modules that are significantly differentially expressed between lung cancer and normal lung tissues, we seek to find those that are specific to either SCLC or NSCLC lung cancer subtype. To do this, we use the module's expression value (the median expression value of all genes inside the module) to classify SCLC and NSCLC cancer samples and plot an ROC curve for each module. The AUC (area under curve) of an ROC ranges from 0 to 1, with 0.5 indicating randomness. AUC of 0 or 1 corresponds to perfect specific in distinguishing the two lung cancer subtypes. In addition, as we try to classify SCLC against NSCLC, an AUC close to 1.0 indicates that the module is upregulated in SCLC compared to that in NSCLC, and if it is close to 0.0, then the module is downregulated in SCLC. We find 10 modules with AUC score greater than 0.9, indicating that these modules are strongly upregulated in SCLC compared to NSCLC. There are also 16 modules with scores smaller than 0.1, suggesting that they are strongly downregulated in SCLC.

After identifying gene modules that have distinct expression pattern between SCLC and NSCLC, we further seek to find genes that are of diagnostic use for distinguishing SCLC and NSCLC. For this purpose, we develop a score function to measure the ability to distinguish the two subtypes of lung cancer for each gene. This score integrates both the cancer type specificity of the module and the coexpression value difference of the gene. The cancer type specificity of a module is simply defined by the absolute value of AUC-0.5. The coexpression value difference of a gene in a given module is determined by the average of the coexpression value difference of the interactions involving this gene in this module. The coexpression value difference for a given interaction is defined by the difference in coexpression pattern of the two interacting genes between SCLC and NSCLC. For example, for two interacting genes A and B, if they are positively coexpressed in SCLC while negatively coexpressed in NSCLC (see Section 2 for the definition of positively or negatively coexpressed gene pairs), then the coexpression value difference will be 1 − (−1) = 2 and vice versa. However, if the two interacting genes are positively or negatively coexpressed in one cancer subtype while randomly coexpressed in another one, then the coexpression value will be 1 − 0 = 1. If both genes are either positively or negatively coexpressed in both cancer types, then the coexpression value difference will be 0. By computing the coexpression value difference of all interactions in the module that involve the gene under test, we can compute the average and use it to represent the coexpression value difference for this gene. Finally, the coexpression value of the gene is multiplied by the cancer type specificity of the module to produce a score for the gene.

**Figure 2:**
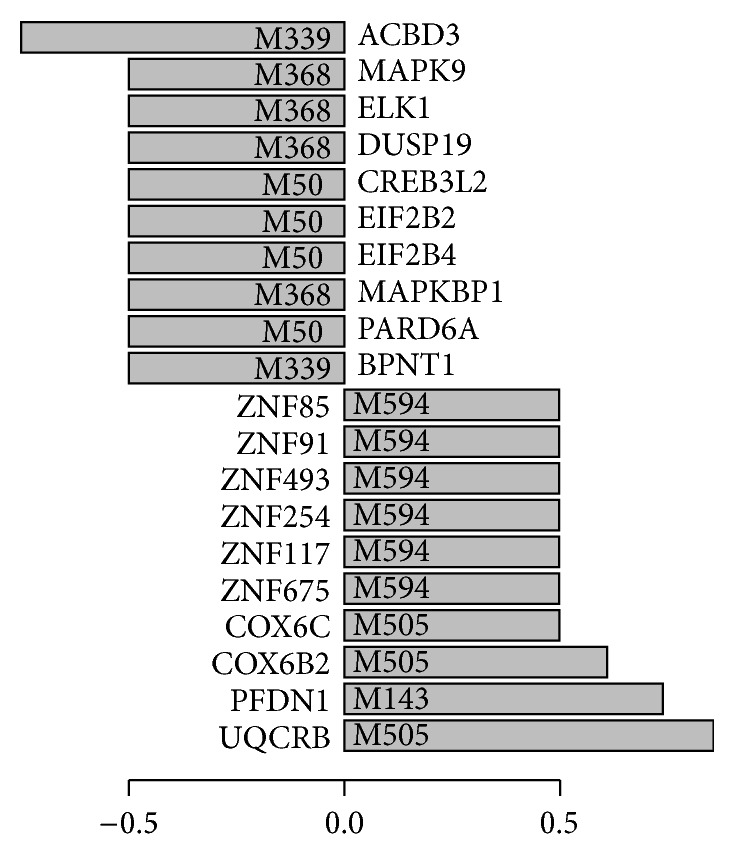
Bar plot for the top 10 upregulated and top 10 downregulated genes in SCLC, compared to that in NSCLC. The bar height of each gene represents the predicted score for the gene that distinguishes SCLC samples from NSCLC samples (see Section 2 for details). Genes with score greater than 0 are upregulated genes, while those with score smaller than 0 are downregulated genes. The IDs of the module where the predicted genes belong are shown inside the bar.

**Figure 3:**
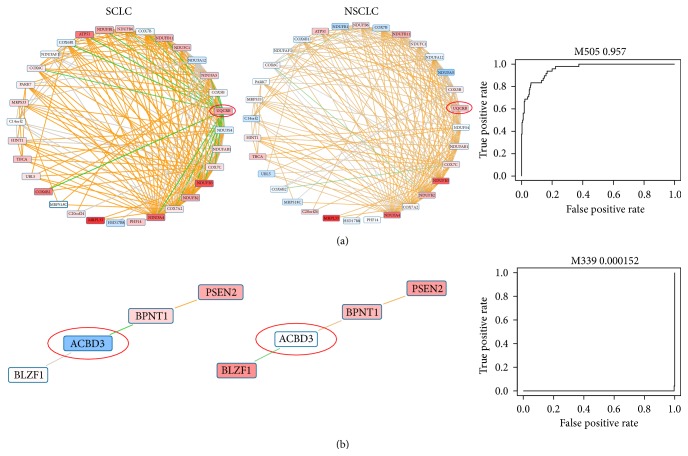
Examples of gene modules and genes that can distinguish SCLC from NSCLC. (a) shows a module that is upregulated in SCLC, while (b) shows a module with only four genes that is downregulated in SCLC. The ROC curve of the two modules is shown at the right of the figure. The color of each gene inside the module represents the expression pattern of this gene between a cancer subtype and the normal lung tissues, with red indicating that it is upregulated in cancer, while blue indicating that it is downregulated. The edge color of each interaction inside the module represents the coexpression information in the corresponding cancer subtypes, with orange indicating positive coexpression while green indicating negative coexpression.

Following the above-described strategy, we have obtained 137 genes with significant power in distinguishing SCLC from NSCLC and show the top 10 upregulated and top 10 downregulated genes in SCLC as candidate genes of diagnostic use in Figure 2. Three upregulated genes in SCLC are from one module named M505 (Figure 3(a)). This module is enriched with mitochondrial related functions. The AUC score for this module is 0.957, indicating that this module is significantly upregulated in SCLC compared to NSCLC. One of the three genes is UQCRB that has the highest score. UQCRB interacts with 18 genes in this module. Among the 18 interactions involving UQCRB, 16 are negatively coexpressed in SCLC while 14 are positively coexpressed in NSCLC. UQCRB is ubiquinol-cytochrome c reductase binding protein and is strongly upregulated in SCLC. It is likely that this gene may be important for the disrupted mitochondrial functions in SCLC. Interestingly, we find that there is a SNP located on UQCRB, rs7827095, which is strongly related to lung disease according to a cohort study [30]. As for genes downregulated in SCLC, ACBD3 has the highest score. ACBD3 is present in a module named M339 that is strongly downregulated in SCLC with an AUC score of 0.000152 (Figure 3(b)). ACBD3 interacts with two genes in this module, which are BPNT1 and BLZF1. Interestingly, ACBD3 and BPNT1 are negatively coexpressed in SCLC, while ACBD3 and BLZF1 are negatively coexpressed in NSCLC. ACBD3 is acyl-CoA binding domain containing 3 protein and is involved in hormone regulation of steroid formation. This gene has been reported to show significant difference between responders (PR) and nonresponders (PD) to gefitinib in NSCLC treatment [31]. Here, our study suggests that ACBD3 is worthy of further exploitation to understand its involvement in the development of NSCLC. The full list of predicted genes is shown in Supplementary Table 1.

### 3.5. Further Validation of the Proposed Method

**Figure 4:**
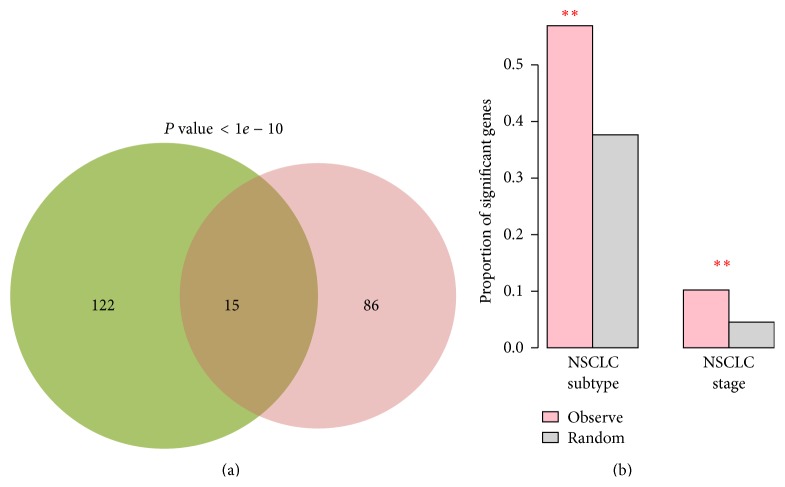
(a) Venn diagram to show the overlap between the genes predicted by using the new NSCLC dataset and those predicted based on the original three datasets. (b) Proportion of genes among the predicted genes that are significant in distinguishing between different subtypes and stages of NSCLC and that are among randomly selected genes. “∗∗” indicate that the observed proportion is significantly higher than random.

To further validate our method, we select a new NSCLC dataset (GSE10245) which has 58 samples (the original dataset includes more than 100 samples) and then repeat the analysis to predict genes that can distinguish SCLC from NSCLC. We obtain 101 genes in which 15 overlaps with the original predictions (Figure 4(a)). The overlap is significantly higher than random (*P* value < 1*e* − 10), indicating that our method is robust to the choice of datasets.

In addition, we inspect whether the predicted genes based on the original datasets can be used to distinguish different stages of lung cancer or the subtypes within either SCLC or NSCLC. The NSCLC dataset actually includes not only stage information, but also the subtypes of NSCLC information. The stage information is 48 IA, 84 IB, 11 IIA, 39 IIB, 51 IIIA, 35 IIIB, and 7 other stages. In this dataset, there are 14 subtypes of NSCLC, including 183 adenocarcinomas, 80 squamous, and 12 other subtypes of NSCLC. Among the 137 predicted genes, we find 78 genes that can distinguish adenocarcinoma from squamous samples based on their expression levels and 14 genes that have significant gene expression variation in at least one stage of NSCLC (see Section 2 for details of the test procedure). The proportion of those genes among the predicted genes is significantly higher than that of randomly selected genes (both *P* values < 0.01) (Figure 4(b)). These results suggest that a significant proportion of our predicted genes may also carry information to distinguish between different stages and subtypes of NSCLC.

## 4. Discussion

Lung cancer is the leading cause for cancer related death worldwide. It can be classified into two main subtypes (SCLC and NSCLC) according to their physiological phenotypes. In this study, we have conducted a computational study to predict molecular biomarkers that are of potential diagnostic use for distinguishing the two types of lung cancer. By collecting gene pairs that are significantly positively or negatively coexpressed in normal lung tissues, SCLC samples, and NSCLC samples and considering their functional associations, we have constructed a lung cancer-specific gene functional association network. After partitioning the network into gene modules, we identify gene modules that have significant expression variation between lung cancer and normal lung tissues. We then compute the specificity of each of these modules in distinguishing SCLC from NSCLC and further identify candidate genes inside the module that have discriminating power for lung cancer subtypes. There are several interesting findings that resulted from this study. First of all, we find that gene modules that are upregulated in both lung cancer subtypes are significantly enriched with glycolysis metabolism, while those downregulated gene modules shared for the two lung cancer subtypes are enriched with phenylalanine metabolism, suggesting that there are common altered metabolisms in the development of both SCLC and NSCLC. Secondly, we find gene modules that are differentially expressed only in one subtype of lung cancer and are enriched with specific functions. For example, gene modules upregulated only in SCLC are enriched with functions in chromatin modeling, while those upregulated in NSCLC are enriched with DNA replication process, suggesting that there may be unique mechanism for the development of specific lung cancer subtypes. Thirdly, we have obtained a list of genes that have significant discriminating power in distinguishing lung cancer subtypes. These genes are identified by integrating both cancer-specificity information and coexpression information and provide novel hypothesis for the development of specific subtype of lung cancer. Finally, although the study is designed for identifying molecular biomarker for distinguishing lung cancer subtypes, the methodology developed here can be readily applied for distinguishing the subtypes of other types of diseases.

## Supplementary Material

The detailed information about the differentially expressed gene modules in lung cancer as well as their functional analysis.

## Figures and Tables

**Table 1:** Summary of datasets used in our study.

GEO ID	Sample type	Sample number	Sample information	Reference
GSE23546	Normal	1349	Nontumor lung tissues from patients with lung cancer	[13]

GSE41271	NSCLC	275	275 tumor specimens from ~1,700 non-small-cell lung cancer specimens collected at the MD Anderson Cancer Center over the years 1997 to 2005	[14]

GDS4794	SCLC	23	23 clinical small-cell lung cancer (SCLC) samples from patients undergoing pulmonary resection	[15]

GSE62021	SCLC	25	25 SCLC tumor tissues	Unpublished

## References

[1] Chen W., Zhang S., Zou X. (2010). Estimation and projection of lung cancer incidence and mortality in China. *Zhongguo Fei Ai Za Zhi*.

[2] Boyle B. L. P. (2008). *World Cancer Report 2008*.

[3] Hoffman P. C., Mauer A. M., Vokes E. E. (2000). Lung cancer. *The Lancet*.

[4] Travis W. D., Brambilla E., Müller-Hermelink H. K., Harris C. C. (2004). *World Health Organization Classification of Tumours: Pathology and Genetics: Tumours of the Lung, Pleura, Thymus and Heart*.

[5] van Meerbeeck J. P., Fennell D. A., De Ruysscher D. K. M. (2011). Small-cell lung cancer. *The Lancet*.

[6] Wistuba I. I. (2007). Genetics of preneoplasia: lessons from lung cancer. *Current Molecular Medicine*.

[7] Thunnissen E., Kerr K. M., Herth F. J. F. (2012). The challenge of NSCLC diagnosis and predictive analysis on small samples. Practical approach of a working group. *Lung Cancer*.

[8] Edwards S. L., Roberts C., McKean M. E., Cockburn J. S., Jeffrey R. R., Kerr K. M. (2000). Preoperative histological classification of primary lung cancer: accuracy of diagnosis and use of the non-small cell category. *Journal of Clinical Pathology*.

[9] Arya S. K., Bhansali S. (2011). Lung cancer and its early detection using biomarker-based biosensors. *Chemical Reviews*.

[10] Zhang H.-H., Zhang Z.-Y., Che C.-L., Mei Y.-F., Shi Y.-Z. (2013). Array analysis for potential biomarker of gemcitabine identification in non-small cell lung cancer cell lines. *International Journal of Clinical and Experimental Pathology*.

[11] Mishra D. K., Creighton C. J., Zhang Y., Gibbons D. L., Kurie J. M., Kim M. P. (2014). Gene expression profile of A549 cells from tissue of 4D model predicts poor prognosis in lung cancer patients. *International Journal of Cancer*.

[12] Karni S., Soreq H., Sharan R. (2009). A network-based method for predicting disease-causing genes. *Journal of Computational Biology*.

[13] Bossé Y., Postma D. S., Sin D. D. (2012). Molecular signature of smoking in human lung tissues. *Cancer Research*.

[14] Sato M., Larsen J. E., Lee W. (2013). Human lung epithelial cells progressed to malignancy through specific oncogenic manipulations. *Molecular Cancer Research*.

[15] Sato T., Kaneda A., Tsuji S. (2013). PRC2 overexpression and PRC2-target gene repression relating to poorer prognosis in small cell lung cancer. *Scientific Reports*.

[16] Szklarczyk D., Franceschini A., Kuhn M. (2011). The STRING database in 2011: functional interaction networks of proteins, globally integrated and scored. *Nucleic Acids Research*.

[17] Davis S., Meltzer P. S. (2007). GEOquery: a bridge between the Gene Expression Omnibus (GEO) and BioConductor. *Bioinformatics*.

[18] Barrett T., Troup D. B., Wilhite S. E. (2007). NCBI GEO: mining tens of millions of expression profiles—database and tools update. *Nucleic Acids Research*.

[19] Smyth G. K. (2005). Limma: linear models for microarray data. *Bioinformatics and Computational Biology Solutions Using R and Bioconductor*.

[20] Sun S., Dong X., Fu Y., Tian W. (2011). An iterative network partition algorithm for accurate identification of dense network modules. *Nucleic Acids Research*.

[21] Kuner R., Muley T., Meister M. (2009). Global gene expression analysis reveals specific patterns of cell junctions in non-small cell lung cancer subtypes. *Lung Cancer*.

[22] Ashburner M., Ball C. A., Blake J. A. (2000). Gene ontology: tool for the unification of biology. *Nature Genetics*.

[23] Liberzon A., Subramanian A., Pinchback R., Thorvaldsdóttir H., Tamayo P., Mesirov J. P. (2011). Molecular signatures database (MSigDB) 3.0. *Bioinformatics*.

[24] Gatenby R. A., Gillies R. J. (2004). Why do cancers have high aerobic glycolysis?. *Nature Reviews Cancer*.

[25] Dionisi M. S., Rubino S. (1995). Asthma associated with small-cell lung cancer. *Supportive Care in Cancer*.

[26] de Castro J., Rodríguez M. C., Martínez-Zorzano V. S., Sánchez-Rodríguez P., Sánchez-Yagüe J. (2014). Erythrocyte Fatty acids as potential biomarkers in the diagnosis of advanced lung adenocarcinoma, lung squamous cell carcinoma, and small cell lung cancer. *American Journal of Clinical Pathology*.

[27] Zienolddiny S., Campa D., Lind H. (2006). Polymorphisms of DNA repair genes and risk of non-small cell lung cancer. *Carcinogenesis*.

[28] Yang P., Bamlet W. R., Ebbert J. O., Taylor W. R., de Andrade M. (2004). Glutathione pathway genes and lung cancer risk in young and old populations. *Carcinogenesis*.

[29] Galluzzo P., Bocchetta M. (2011). Notch signaling in lung cancer. *Expert Review of Anticancer Therapy*.

[30] Ong B. A., Li J., McDonough J. M. (2013). Gene network analysis in a pediatric cohort identifies novel lung function genes. *PLoS ONE*.

[31] Fan J., Liu J., Culty M., Papadopoulos V. (2010). Acyl-coenzyme A binding domain containing 3 (ACBD3; PAP7; GCP60): an emerging signaling molecule. *Progress in Lipid Research*.

